# High levels of T lymphocyte activation in *Leishmania*-HIV-1 co-infected individuals despite low HIV viral load

**DOI:** 10.1186/1471-2334-10-358

**Published:** 2010-12-20

**Authors:** Joanna R Santos-Oliveira, Carmem BW Giacoia-Gripp, Priscilla Alexandrino de Oliveira, Valdir S Amato, Jose Ângelo L Lindoso, Hiro Goto, Manoel P Oliveira-Neto, Marise S Mattos, Beatriz Grinsztejn, Mariza G Morgado, Alda M Da-Cruz

**Affiliations:** 1 Laboratório Interdisciplinar de Pesquisas Médicas, Instituto Oswaldo Cruz -FIOCRUZ. Av. Brasil 4365. Rio de Janeiro, CEP 21040-360, Brazil; 2 Laboratório de Aids e Imunologia Molecular; Instituto Oswaldo Cruz - FIOCRUZ, Av. Brasil 4365. Rio de Janeiro, CEP 21040-360, Brazil; 3 Hospital-Dia Profa. Esterina Corsini, Hospital Universitário, Universidade Federal de Mato Grosso do Sul (UFMS). Mato Grosso do Sul, CEP 79070-900, Brazil; 4 Serviço de Doenças Infecciosas e Parasitárias, Faculdade de Medicina, Universidade de São Paulo, São Paulo, Brazil. CEP 05403-010, São Paulo, Brazil; 5 Instituto de Medicina Tropical de São Paulo - Universidade de São Paulo, São Paulo, CEP 05403-010, Brazil; 6 Instituto de Pesquisa Clínica Evandro Chagas, IPEC - FIOCRUZ, Av. Brasil 4365. Rio de Janeiro, CEP 21040-360, Brazil

## Abstract

**Background:**

Concomitant infections may influence HIV progression by causing chronic activation leading to decline in T-cell function. In the Americas, visceral (AVL) and tegumentary leishmaniasis (ATL) have emerged as important opportunistic infections in HIV-AIDS patients and both of those diseases have been implicated as potentially important co-factors in disease progression. We investigated whether leishmaniasis increases lymphocyte activation in HIV-1 co-infected patients. This might contribute to impaired cellular immune function.

**Methods:**

To address this issue we analyzed CD4^+ ^T absolute counts and the proportion of CD8^+ ^T cells expressing CD38 in *Leishmania*/HIV co-infected patients that recovered after anti-leishmanial therapy.

**Results:**

We found that, despite clinical remission of leishmaniasis, AVL co-infected patients presented a more severe immunossupression as suggested by CD4^+ ^T cell counts under 200 cells/mm^3^, differing from ATL/HIV-AIDS cases that tends to show higher lymphocytes levels (over 350 cells/mm^3^). Furthermore, five out of nine, AVL/HIV-AIDS presented low CD4^+ ^T cell counts in spite of low or undetectable viral load. Expression of CD38 on CD8^+ ^T lymphocytes was significantly higher in AVL or ATL/HIV-AIDS cases compared to HIV/AIDS patients without leishmaniasis or healthy subjects.

**Conclusions:**

*Leishmania *infection can increase the degree of immune system activation in individuals concomitantly infected with HIV. In addition, AVL/HIV-AIDS patients can present low CD4^+ ^T cell counts and higher proportion of activated T lymphocytes even when HIV viral load is suppressed under HAART. This fact can cause a misinterpretation of these laboratorial markers in co-infected patients.

## Background

*Leishmania*/HIV-1 co-infection has been considered an emerging disease mainly due to the expansion of the AIDS epidemic over leishmaniasis endemic areas and vice-versa [1]. Visceral leishmaniasis associated-HIV/AIDS is well known as an opportunistic disease especially in the Mediterranean basin [1]. However, an increasing number of reports of tegumentary leishmaniasis-HIV/AIDS patients underlines the importance of this additional association [2-6]. In America, vector sandflies and *Leishmania *species that cause American visceral leishmaniasis (AVL) and tegumentary leishmaniasis (ATL) differ from those observed in other endemic regions around the world. This could result in the particular clinical features observed in HIV-co-infected patients in the New World [3,7]. In Brazil, both *Leishmania *and HIV infection are endemic and this co-infection is becoming an important public health problem [1,7].

The clinical outcome of both AVL and ATL is dependent on the *Leishmania*-specific immune response. This influence can be exerted in many different ways. AVL is a systemic infection affecting lymphoid organs; it is associated with parasite antigens driving an impaired T-cell response leading to a depletion of bone marrow as well as systemic CD4^+ ^and CD8^+ ^T lymphocytes. Profound immunosuppression is the consequence [8-11]. In contrast, ATL is a benign disease which affects skin and mucous membranes. In these ATL patients, the specific immune response is preserved though not well regulated, leading to tissue damage [10,12]. Even considering the differences in the clinical and immunopathogenesis of those AVL and ATL, T lymphocyte function is affected in both diseases, and the clinical cure is achieved by restoration of an adequate effector immune response [9-12].

Earlier studies have shown that *Leishmania *infection can promote HIV-1 replication *in vitro *[13] and *in vivo *[14]. Beside this, HIV-1 infection impairs macrophage ability to control intracellular protozoans, enabling *Leishmania *growth [15,16]. While highly effective antiretroviral therapy (HAART) has been shown to decrease significantly the incidence of leishmaniasis in HIV-infected patients [17,18], it does not seem to prevent relapses [19]. This suggests that maintenance of the parasite can impact the pathogenesis of both diseases.

Low lymphocyte recall responses after parasite stimulation but preserved IFN-γ production in ATL-HIV-AIDS patients were already demonstrated [3]. On the other hand, high type 2 cytokines levels are observed in HIV/AIDS associated AVL, potentially favoring the dissemination of both the virus and *Leishmania *[14]. Moreover, increased levels of CCR5 molecules on CD3^+ ^lymphocytes were also observed in AVL/HIV-AIDS [20], suggesting that *Leishmania *infection could favor HIV-1 entry into its target cells through this receptor. Immunological studies reporting *Leishmania*/HIV co-infection generally involve patients with active disease [3,14,20,21], but the mechanisms that maintain leishmaniasis in remission, and by consequence the factors that predispose the parasite reactivation are poorly understood.

Chronic activation of the immune system has been considered an important mechanism related to declining lymphocytic functions and has become an important tool for monitoring HIV-1 infected patients [22]. The proportion of CD8^+ ^T cells that express CD38^+^, a surrogate of activation marker, can predict the subsequent decline of the CD4^+ ^T cell population [23] and was correlated with high viral load levels in HIV infected individuals [24]. Actually, the degree of cellular activation, evidenced by the co-expression of CD38 and HLA-DR on T lymphocytes has been considered an important predictor of disease progression [25]. Concomitant infections can exert an impact on viral replication by contributing to an increase in T lymphocyte activation already seen in HIV patients [26]. In accordance with this, co-infection with HIV and tuberculosis results in high levels of CD38 on CD8^+ ^T cells [27]. Nevertheless, viral infections concomitant with HIV-1 infection seem to have different effects on T lymphocyte activation. Recent evidence showed that Hepatitis C virus/HIV-1 co-infected patients presented elevated immune activation levels, despite effective antiretroviral therapy [28]. On the other hand, GB virus type C replication has been associated to lower T cell activation, which has considered a protective mechanism involved in the HIV-1 disease progression [29]. No influence of human herpesvirus replication on cell activation has been observed [30]. However, the impact of *Leishmania *infection on T cell perturbation due to HIV-1 is still unclear." We hypothesized that *Leishmania *infection could be an additional factor for lymphocyte activation and lead to impaired effect or immune function, favoring parasite replication and, consequently, frequent relapses. Therefore, we investigated whether leishmaniasis could impact T lymphocytes activation in HIV-1 co-infected patients through the analysis of CD4^+ ^T absolute counts and of the proportion of CD8^+ ^T cells expressing CD38 in *Leishmania*/HIV co-infected patients recovered after anti-leishmanial therapy.

## Methods

### Patients and Subjects

Seventeen HIV-*Leishmania *co-infected patients (AVL-HIV/AIDS - 9 cases and ATL-HIV/AIDS - 8 cases) were enrolled for this study. Patients were in the remission phase, minimum six months after the end of their anti-*Leishmania *treatment and had no signs or symptoms of active leishmaniasis (Table 1). Sixteen HIV-1/AIDS cases, without previous leishmaniasis paired with co-infected patients by viral load (HIV RNA copies/mL: < 400, > 400 to 10.000 and over > 10,000 copies/mL) and eight healthy volunteers were included as controls. HIV/AIDS patients were receiving antiretroviral therapy for at least one year, according to the Brazilian guidelines. Informed consent was obtained from all participants. The study was approved by the Fundação Oswaldo Cruz and IPEC Ethical Committees.

**Table 1:** Clinical and laboratorial characteristics of HIV-1 co-infected leishmaniasis patients and control groups.

Parameters	AVL/HIV-1 patients (n = 9)	ATL/HIV-1 patients (n = 8)	HIV-1 infected* (n = 16)	Healthy subjects (n = 8)
Age, years, (median)	38 (35-50)^a^	44 (38-54)^b^	39 (33-49)^c^	26 (25-30)^abc^
Male sex, n, (%)	9 (100)^d^	8 (100)^e^	15 (94)^f^	4 (50)^def^
CD4^+^T Cell count, cells/mm^3^	62 (52-127)^g^	404 (294-597)^h^	380 (223-450)^i^	1,106 (957-1,300)^ghi^
Current AIDS diagnosis, Number of cases (%)	9 (100)	6 (75)	8 (50)	----
Time of clinical remission of leishmaniasis, months	8 (6-12)	11 (7.5-14)	----	----
Patients with undetectable viremia, (%)	5 (55.6)	4 (50)	9 (56.2)	----
Viral load levels of patients with detectable viremia, copies/mL	142,240 (24,025-279,321)	6,200 (2,012-78,176)	12,010 (2,000-136,625)	----

N = number, AVL - American visceral leishmaniasis, ATL - American tegumentary leishmaniasis *HIV-1 infected patients without leishmaniasis Note: Data are median (interquartile range) values, except for current AIDS diagnosis.

Patients × Health subjects (a - p < 0.001; b/c - p < 0.01; d/e/f × def - p < 0.05), g/h/i × ghi - p < 0.001; g × h - p < 0.01; g × i - p < 0.01

### Immunologic and virologic assessments

To determinate the absolute counts of CD4^+ ^T and CD8^+ ^T lymphocytes, a BD Tritest^® ^monoclonal antibody specific for CD4/CD8/CD3 conjugated to FITC, PE and PerCP, respectively and BDTrue Count^® ^reagent kit was used according to the manufacturer's instructions (BD Biosciences, Franklin Lakes, NJ, USA). Samples were acquired using a FACSCalibur^® ^(BD, USA) and analysed by Multiset^® ^software (BD, USA). Plasma HIV-1 RNA levels were quantified using the branched DNA assays (Siemens, Versant HIV-1 RNA 3.0, Tarrytown, NY, USA). The lower limit of detection for this assay was 50 copies/mL.

### Obtainment of peripheral blood mononuclear cells (PBMC) and immune activation

PBMC were separated by centrifugation over a gradient of Ficoll-Hypaque (Histopaque 1077; Sigma Chemical Company, St. Louis, MO, USA) and ressuspended in phosphate-buffered saline (PBS) containing 0,1% of sodium azide (Sigma, USA). PBMCs were adjusted to10^5 ^cells and labeled with anti-CD8 FITC plus anti-CD38 PE monoclonal antibodies (BD Simultest™, BD Biosciences, San Jose, CA, USA). After incubation, the cells were fixed with PBS plus 1% paraformaldehyde. At least 10,000 events were acquired using a FACSCalibur and phenotypic analysis was carried out by using CellQuest™software (BD). Although the validated marker of CD8^+^T cell activation is the co-expression of CD38 and HLA DR, we used the detection of CD38 in CD8^+ ^as a surrogate of this validated marker [25]. CD38 positivity was determined on CD8^+ ^T cell populations inside the gate previously established for TCD3^+ ^and expressed as a percentage of CD38 in CD8^high^.

### Statistical analysis

The values were expressed as medians and interquartile range. Statistical analysis was performed using the Mann-Whitney U test (GraphPad Prism, version 4.0, San Diego, CA, USA). Multivariate linear regression analysis (SPSS software, version 9.0) was used to determine influence that of intervenient factors on the percentage of CD8^+ ^T cells expressing CD38 (dependent variable). Absolute counts of CD4^+ ^T cells, viral load (undetectable or detectable) and *Leishmania *infection (presence or absence) were considered as independent variables. Statistically significant differences were considered when *p *< 0.05.

## Results

**Figure 1:**
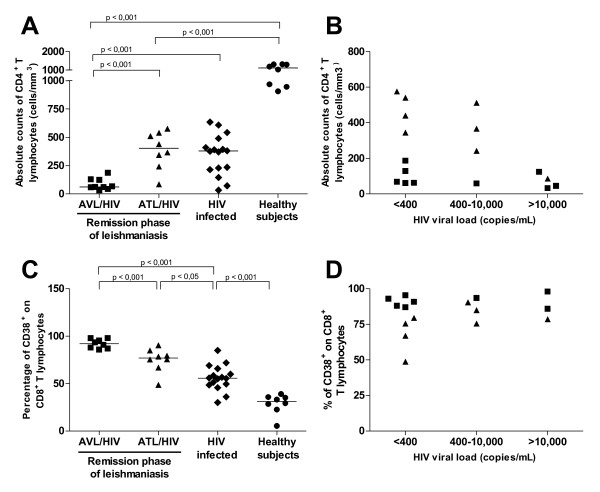
**Relationship between lymphocyte immune status and plasmatic HIV-1 viral load in *Leishmania*/HIV-1 co-infected patients during remission phase of leishmaniasis**. **A**. Absolute counts of CD4^+ ^T lymphocytes. **B**. Absolute counts of CD4^+ ^T lymphocytes and viral load levels. **C**. Levels of CD38 expression on CD8^+ ^T lymphocytes. **D**. CD38 expression on CD8^+ ^T lymphocytes and viral load levels. American visceral leishmaniasis (AVL)/HIV-AIDS patients (solid squares), American tegumentary leishmaniasis (ATL)/HIV-AIDS patients (solid triangles), HIV-1 infected adults without leishmaniasis (HIV infected, solid diamonds) and healthy subjects (solid circles). Each point represents one subject. The horizontal bars express median.

Clinical and laboratory characteristics are depicted in Table 1 and 2. AVL-HIV/AIDS patients in clinical remission showed significantly (p < 0.001) lower numbers of CD4^+ ^T lymphocytes in comparison to HIV-1 infected patients without leishmaniasis, (Figure 1A and Table 1). In contrast, CD4^+ ^T counts in ATL-HIV/AIDS patients were similar to HIV-1 infected patients without leishmaniasis (Figure 1A). Both types of co-infected patients and the HIV-1 infected patients without leishmaniasis had much lower levels of CD4^+ ^T counts when compared to healthy donors (p < 0.001, Figure 1A).

**Table 2:** CD4+ T cell counts and viral load levels of HIV-AIDS associated leishmaniasis patients (visceral or tegumentary) and HIV-1 infected control group.

Patient's Number	CD4^+ ^T cells counts(cells/mm^3^)	Viral load levels (copies/mL)
**VL/HIV-AIDS**		
**1**	78	< 400
**2**	59	5,810
**3**	33	316,402
**4**	129	< 400
**5**	61	< 400
**6**	45	242,240
**7**	187	< 400
**8**	124	42,240
**9**	68	< 400
**TL/HIV-AIDS**		
**1**	367	1,700
**2**	440	< 400
**3**	512	2,324
**4**	86	146,351
**5**	541	< 400
**6**	345	< 400
**7**	576	< 400
**8**	242	10,000
**HIV-1 infected**		
**1**	146	136,625
**2**	543	< 400
**3**	215	< 400
**4**	34	12,010
**5**	230	253,761
**6**	609	< 400
**7**	391	< 400
**8**	491	< 400
**9**	377	< 400
**10**	73	1,750
**11**	371	10,000
**12**	236	< 400
**13**	635	< 400
**14**	382	2,000
**15**	394	55,239
**16**	410	< 400

HIV-1 viral load in AVL/HIV-AIDS was not correlated with CD4^+ ^T cell count, since low or undetectable levels of HIV-1 were seen even in patients with very low CD4^+ ^T cells counts (Figure 1B). By contrast, ATL/HIV-AIDS patients in clinical remission had higher CD4^+ ^T cell numbers and exhibited the lowest HIV plasma viral load (Figure 1B).

HIV-1 co-infected individuals (AVL and ATL), had a significantly higher percentage of CD8^+ ^T cells that expressed CD38 when compared to HIV-1 infected patients without leishmaniasis (median 55.8%; [49% - 64%], p < 0.05) (Figure 1C). However, the frequency of CD38 expressed by the CD8^+ ^T cell subset was much higher in AVL/HIV-AIDS cases (median 93.5%, [87%-97.9%]) than in patients with ATL/HIV-AIDS (median 77%, [69%-83.6%], p < 0.001). Healthy blood donors showed the lowest levels of CD8^+ ^T cell activation (CD38^+ ^on CD8^+ ^T cells = median 31.2%; [24%-35.6%], p < 0.001) (Figure 1C). There was no correlation between the percentages of CD38 on CD8^+ ^T cell and plasma HIV-1 viral load (Table 3), since high levels of activation were observed despite viral load levels in the co-infected patients (Figure 1D). HIV-1 infected controls presented a positive correlation between the viral load and cellular activation (p = 0.01, r = 0.60, data not show).

**Table 3:** Multivariate linear regression analysis to evaluate the association between T cell activation (CD38+ on CD8+ T lymphocytes) and independent variables in leishmaniasis and HIV-1 co-infected patients.

Independent variables	Dependent variable		
	Percentage of CD38^+ ^on CD8^+ ^T lymphocytes		
	**Coef^1^**	**SE^2^**	***P***
*Leishmania *infection (presence or absence)	24.88	4.63	0.000011
CD4^+ ^T cell count, cells/mm^3^	-0.02	0.013	0.13
Viral load levels (detectable or undetectable)	4.12	4.85	0.39

1 Coef - Correlation coefficient, 2 SE - Standard error

Obs. HIV-1 infected patients were also included in this analysis.

A multivariate statistical analysis showed a significant positive correlation between *Leishmania *infection and the levels of CD38 on CD38 T lymphocytes (p < 0.001), independently of CD4^+ ^T counts and HIV viremia (Table 3). A covariance was not performed because of the low number of ATL and AVL patients.

## Discussion

*Leishmania *can persist in the host after treatment and may further reactivate under immunossupression [1]. A host parasite dynamic equilibrium does not seem to be reached in HIV co-infected patients probably because immunosupressed patients are not able to mount an efficient T-cell response to control *Leishmania*, resulting in frequent relapses especially in AVL but also in ATL [1,5]. We found that despite clinical remission of protozoan disease, AVL co-infected patients were more severely immunossupressed than ATL co-infected patients. Furthermore, lymphocyte activation status was higher in both groups of co-infected patients than in HIV-1-infected patients without leishmaniasis. Our results suggest that *Leishmania *infection increases the degree of the immune system activation in individuals concomitantly infected with HIV-1. This phenomena can affect immune effector function and may constitute an additional mechanism for explain relapses.

Both AVL and ATL tend to emerge as opportunistic diseases in HIV infected patients without leishmaniasis especially in those presenting with CD4^+ ^T cell levels below 350 cells/mm^3 ^[1,4,5,31,32]. In this study, AVL/HIV-AIDS patients presented very low CD4^+ ^T cell levels during remission of clinical symptoms after successful anti-leishmanial therapy and under HAART use. This was not observed in ATL/HIV-AIDS. The low CD4^+ ^T cell counts observed in AVL co-infected patients cannot be solely explained by lymphocyte depletion related to HIV-1 replication, since low CD4^+ ^T cell counts were observed in AVL/HIV-AIDS patients with undetectable or low viral load levels. Additionally, CD4 T cell depletion of bone marrow caused by both HIV infection [33] and AVL [9,11] was already demonstrated and may lead to deficiencies in the input of new lymphocytes into the periphery. These factors could explain why AVL/HIV-AIDS patients already had CD4 cell counts lower than 200 cells/mm^3 ^at the beginning of anti-leishmanial therapy (data not shown), which additionally could contribute to the maintenance of low levels during remission phase. By contrast, the immunopathogenesis of ATL/HIV-AIDS may not be related to systemic imunossuppression since ATL is more restricted to the tegument and the draining lymph nodes. In this connection, these ATL/HIV-AIDS patients in remission phase apparently recovered their lymphocyte levels (data not shown) since they have much higher CD4^+ ^T cell counts than those previously observed in patients with active disease [3-5].

In the present study we have shown that leishmaniasis/HIV-AIDS patients displayed elevated levels of CD38 on CD8^+ ^T cells levels in relation to HIV-1 infected patients without leishmaniasis. This implies that concomitant *Leishmania *infection could contribute to enhance the activation induced by HIV antigens as has been described in tuberculosis [27] and hepatitis C virus [28]. The finding of high CD38 levels positively correlated with *Leishmania *infection reinforces the idea that leishmaniasis can be a cofactor to of this heightened activation status. It is known that high activation levels are inversely proportional to the CD4^+ ^T cell levels, suggesting that activation could also contribute to the T cell depletion [22,25,34]. Interestingly, CD38 levels observed in co-infected patients were much higher than those seen in cured AVL and ATL patients without HIV/AIDS (unpublished data). This result raises the possibility that parasite persistence might have a role in this activation process, since released *Leishmania *antigens can stimulate lymphocytes. The frequent relapses described in AVL/HIV-AIDS, which occur more frequently than in ATL/HIV-AIDS patients, could be evidence of this reduced ability to maintain the parasite under control due to the impaired immune function [4,19]. Viscerotropic *Leishmania *species in the bone marrow promote immunological damage to a greater extent than dermotropic parasites do in the skin [4,11].

This could be, at least in part, why activation levels were higher in AVL/HIV-AIDS than in ATL/HIV-AIDS patients.

## Conclusion

Taken together, our results point to co-infection with leishmaniasis as a factor that is likely to increase the severity of immunodeficiency caused by HIV, especially in AVL/HIV-AIDS patients. The low T CD4 cell counts among those patients with AVL/HIV-AIDS after remission, even in the absence of detectable viral load may have important implications for the HAART monitoring. Furthermore, the prognostic value of CD38 expression on CD8^+ ^T cells might not be useful in predicting HIV-1 progression in co-infected patients [35] since these molecules levels stay elevated after clinical remission of leishmaniasis. To our knowledge no previous study has evaluated the degree of immune activation in *Leishmania*/HIV-1 co-infected patients. Further studies evaluating the consequences of high levels of cellular activation will be of great interest as immunological abnormalities in T lymphocyte function can impact the clinical course of both infections.

## Competing interests

None of the authors has any potential financial conflict of interest related to this manuscript.

## Authors' contributions

JRSO, AMC participated in the conception and design of the study; analysis and interpretation of data, drafting the paper, CBGG and MGM - participated in the conception and design of the study, analysis and interpretation of data, or substantially revising it. PAO, VSA, JALL, HG, MPON, MSM and BG - participated in the recruitment and follow up of the patients of this study. All authors read and approved the final manuscript.

## Pre-publication history

The pre-publication history for this paper can be accessed here:

http://www.biomedcentral.com/1471-2334/10/358/prepub
